# Do Women Prefer More Complex Music around Ovulation?

**DOI:** 10.1371/journal.pone.0035626

**Published:** 2012-04-25

**Authors:** Benjamin D. Charlton, Piera Filippi, W. Tecumseh Fitch

**Affiliations:** 1 Department of Cognitive Biology, University of Vienna, Vienna, Austria; 2 Department of Philosophy and Music, University of Palermo, Palermo, Italy; Centre national de la recherche scientifique, France

## Abstract

The evolutionary origins of music are much debated. One theory holds that the ability to produce complex musical sounds might reflect qualities that are relevant in mate choice contexts and hence, that music is functionally analogous to the sexually-selected acoustic displays of some animals. If so, women may be expected to show heightened preferences for more complex music when they are most fertile. Here, we used computer-generated musical pieces and ovulation predictor kits to test this hypothesis. Our results indicate that women prefer more complex music in general; however, we found no evidence that their preference for more complex music increased around ovulation. Consequently, our findings are not consistent with the hypothesis that a heightened preference/bias in women for more complex music around ovulation could have played a role in the evolution of music. We go on to suggest future studies that could further investigate whether sexual selection played a role in the evolution of this universal aspect of human culture.

## Introduction

Although much has been written about the origins of music we still understand little about how this pervasive aspect of human culture evolved [1]–[3]. One of the main problems is that music, unlike language, has no obvious adaptive function. Current theories include the notion that music is a non-adaptive by-product of speech [4] or the auditory system in general [5], but also that it serves adaptive functions in the contexts of social group cohesion [6] and mother-infant song [7]. Another long standing theory holds that music is a candidate for sexual selection [8], [9]. Somewhat surprisingly though, no empirical studies have attempted to test this hypothesis. Indeed, sex differences in musical processing appear to exist [10], [11] and the propensity of men to produce music, even in cultures where women are freely allowed to do so, suggests that music has a role in sexual courtship [9], [12]. Moreover, musical ability appears to reflect qualities that could be used to discriminate between potential mating partners [13], and the ability to produce complex musical sounds might reveal mental and physical skills that are relevant in a mate choice context, such as the capacity of an individual to learn complex behaviours and the possession of fine motor and neural control. Furthermore, if women have biased sensitivities for increased musical complexity when conception is most likely, ancestral males could have exploited this during sexual courtship [14].

Indeed, women are more sensitive around ovulation to many cues involved in courtship [15], and female performance in music listening tasks is dependent on their position in the menstrual cycle, with the right hemisphere (involved in music perception) appearing to be favoured when oestrogen levels are low [16]. Since oestrogen levels are low at ovulation, which is the peak time for conception [17], it is possible that enhanced female musical appreciation occurs at this time, and this could result in a heightened preference for more complex music. To our knowledge, however, while several studies have investigated the relationship between musical complexity and preferences [18]–[27], none have considered how female preferences for different levels of musical complexity vary across their reproductive cycle.

Here we investigate whether women's preferences for musical complexity vary between low and high fertility stages of their menstrual cycle. To this end, we first ran experiments to confirm that women perceived our computer-generated musical stimuli as differing in complexity, and then presented women with musical stimuli representing different levels of complexity at low and high fertility stages of their reproductive cycle, using ovulation predictor kits to precisely determine peak fertility. Our hypothesis is that women will give their highest preference ratings for musical pieces perceived as being more complex around ovulation. Of the current theories of music evolution [2] only the sexual selection hypothesis predicts an effect of female reproductive stage on complexity-based preferences. Accordingly, if women's preferences for complex music were heightened during high fertility days of their menstrual cycles, this would constitute strong evidence that sexual selection played a role in the evolution of music.

## Materials and Methods

### Ethical statement

The University of Vienna ethics committee approved the work. All participants signed informed consent forms before participating in the experiments and were paid or received course credits in exchange for their participation.

### Participants

The participants for our experiments were 40 female students (aged 18–45 years: mean ± SD = 27.9±6.2 years) from the University of Vienna, Austria. Participants were asked to report whether they had ever studied music or played an instrument, and if so for how many years. Using a coded anonymous survey, all participants verified that they were not taking hormonal contraceptives and that they were not currently pregnant or breast-feeding. In addition, subjects provided information about the first and last day of their current menstrual cycle, and whether their cycle was regular or not. Only naturally cycling women with regular cycles were included in the experiment.

### Musical stimuli

**Figure 1:**
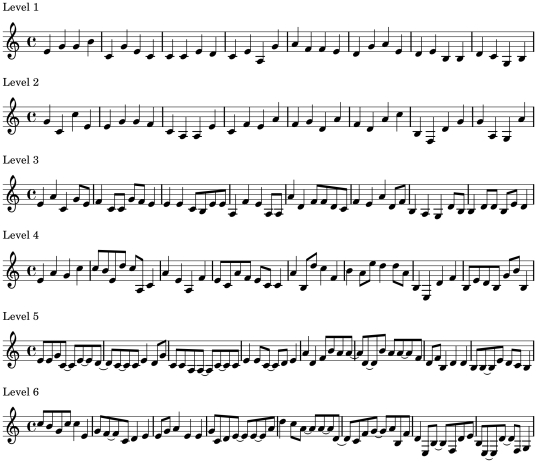
A musical score to illustrate the different levels of complexity for the melody lines of our stimuli. The musical sequences all modulate through two bars of C major, A minor, D minor and G7 major, respectively.

The musical stimuli were created using purpose-built scripts in SuperCollider Version 3.3.1 (http://supercollider.sourceforge.net) and a Markov model based approach. In order to randomly generate the stimuli within certain rules two Markov chains were embedded in the SuperCollider scripts: the first chain operated on all the odd numbered notes (1st, 3rd, 5th 7th etc.) and selected one note from the arpeggio of a given key (e.g., going up an octave in C major this would be C, E, G, C); the second chain operated on all even numbered notes in the melody (2nd, 4th, 6th, etc.) and selected from either 7 or 14 notes of the diatonic scale (see later section). Each musical sequence consisted of eight bars that modulated though two bars of C major, A minor, D minor and G7 major, respectively (see figure 1), and the transition tables for both Markov chains ensured that more widely spaced notes were less likely to follow each other. In addition, by selecting notes from the arpeggio every other beat, each uniquely generated melody was centred on a given key. Importantly, however, by switching between the two Markov chains every other beat, our stimuli still contained a mixture of small and large pitch intervals, leading to the type of moderately predictable and yet moderately surprising melody known to be optimally preferred by listeners [19], [22], [28].

Six different levels of complexity were created: firstly, by varying the number of potential pitch-duration combinations in our melodies, and then by introducing an element of melodic syncopation. Our simplest melodies were constructed using 7 notes of equal duration from the diatonic scale (figure 1, level 1, and Audio S1). For a given key all 7 notes of the major scale were used (for C major: C, D, E, F, G, A and B). More complex melodies were constructed using an additional 7 potential notes from the diatonic scale (figure 1, level 2, and Audio S2), the three immediately below the tonic of a given scale and the four immediately above the 7th note (in C major: G, A, B, C, D, E, F, G, A, B, C, D, E, F). This ensured that the central pitch value for each note range remained roughly the same across conditions whilst expanding the potential note range. To further increase the complexity of melodies constructed using 7 and 14 potential notes we used two different potential note durations, equivalent to a crochet and quaver in musical terminology (figure 1, levels 3 and 4, and Audio S3 and S4, respectively). Finally, in order to create yet higher levels of complexity we introduced an element of melodic syncopation by scrambling a rhythm pattern that consisted of six quavers and five crochets every two bars, and overlaying this pattern onto the notes produced by the Markov chains (figure 1, levels 5 and 6, and Audio S5 and S6, respectively). The amount of syncopation in each eight bar melody was quantified using a metric originally devised by Longuet-Higgins and Lee [29] and subsequently adapted by Fitch and Rosenfeld [30]; giving us mean ± SD syncopation values of 12.9±3.6 and 14.6±5.4 for the melodies comprising levels 5 and 6, respectively.

Previous studies show that increasing potential pitch-duration combinations and introducing melodic syncopation both serve to increase the perceived complexity of musical stimuli [20], [21]. Accordingly, levels 1–6 were intended to represent increasing levels of complexity (see figure 1, and Audio S1, S2, S3, S4, S5, and S6). Melodies were imported into GarageBand (www.apple.com) as MIDI files and, to make the stimuli sound more like a short musical piece, pad chords (Orchestral Strings' MIDI instrument) and a simple 4/4 rhythm were added. The tempo of the stimuli was set to 120 Beats Per Minute and the sequences were saved as AIFF files (44.1 kHz sampling rate, 16 bits amplitude resolution).

### Stimulus presentation

Participants were seated in a quiet room and presented with one of 10 unique stimulus sets, each consisting of four exemplars from each of the six complexity levels. Subjects wore Sennheiser HD 520 headphones and custom software in Python v 2.6 (written by WT Fitch and BD Charlton) was used to present the stimuli in random order and collect mouse-click responses. For all the experiments, participants were first of all presented with six musical sequences representing each of the six different levels of complexity, in order to familiarize them with the experimental protocol and the stimuli.

Two separate psychoacoustic experiments were conducted. In the first, 20 female subjects were asked to rate the 24 musical sequences in a stimulus set for complexity on an 11-point Likert scale. The software interface displayed rating buttons numbered 0 to 10 from left to right, with 0 labelled ‘least complex’ and 10 labelled ‘most complex’. This allowed us to confirm that our stimuli were subjectively rated as differing in complexity, and categorize the six different complexity levels according to perceived complexity. Our second experiment consisted of two parts that were timed to coincide with low and high fertility stages of the menstrual cycle of a further 20 women. Low fertility sessions occurred around 5 days (mean ± SD = 5.1±2.9) before the onset of the next menstruation (confirmed retrospectively). The high fertility sessions were conducted 0–2 days (mean ± SD = 0.3±0.6) after subjects showed a surge in luteinizing hormone (LH), as revealed by an unmarked urine test (Clearblue digital ovulation test: http://www.clearblueeasy.com/clearblue-easy-digital-ovulation-test.php). An LH surge typically precedes ovulation by 24–48 hours [31], thus all subjects were very near the onset of ovulation during their high fertility session.

We used different subjects for this second experiment to eliminate any potential artefacts that might arise from subjects' notions about relationships between complexity and liking ratings [19]. For each session (low and high fertility) subjects were asked to rate how much they liked each of the 24 musical sequences in a stimulus set on a software interface 11-point Likert scale labelled ‘least liked’ (0) to ‘most liked’ (10), and for each subject the low and high fertility sessions were conducted at roughly the same time of day (within 1 hour). The order of the low and high fertility sessions was counterbalanced across subjects, and each subject received the same stimulus set for both sessions, but in a different randomised order.

### Statistical analysis

Linear Mixed Models (LMM's) fitted with maximum likelihood estimation were used for the analysis. For each LMM subject identity was entered as a random factor. In the first LMM we verified the presumed relationship between complexity ratings and complexity levels 1–6: in this model each subject's average complexity rating for the six complexity levels was entered as a dependant variable, complexity level 1–6 was entered as a fixed factor independent variable, and subject age (age), years of formal musical training (musical expertise), and menstrual cycle day normalised to a 28-day cycle (cycle day) were entered as covariates. Cycle day was normalised using each subject's current cycle length in days (calculated using the first and last day of each subject's current menstruation cycle), dividing 28 by the cycle length to create a correction factor, and then multiplying each woman's day in the cycle at the time of the experiment by this correction factor [32], [33]. For example, a women with a 28-day average cycle length would have her current cycle day multiplied by 28/28 = 1 (not corrected), a women with an average cycle length of 40 days would have her current cycle day multiplied by 28/40 = 0.7 and hence, reduced. Pair-wise comparisons with Bonferroni adjustments allowed us to determine whether subjects rated the six different levels of musical complexity as significantly differing in complexity, and group the levels according to their perceived complexity.

For the second experiment, a separate LMM investigated whether liking ratings differed according to reproductive stage (low fertility versus high fertility) and perceived musical complexity. In this model, each subject's average liking rating for the different complexity conditions was entered as a dependant variable, with reproductive stage and complexity condition entered as fixed factor independent variables. Subject age and years of formal musical training were again entered as covariates. All statistical analyses were conducted using SPSS version 19 for Mac OS X, and significance levels were set at 0.05.

## Results

### Complexity ratings

**Figure 2:**
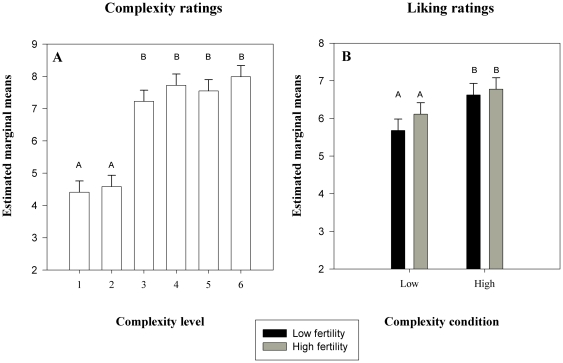
Estimated marginal means ± SE of women's responses to the musical stimuli. Complexity ratings for the different complexity levels 1–6 (A), and liking ratings for low and high musical complexity at the two cycle stages (B) are shown. Mean responses sharing the same letter are not significantly different.

A significant main effect of complexity level on complexity ratings was revealed (*F*_5, 100_ = 26.22, *p*<0.001), confirming that our stimuli were perceived as differing in complexity (see figure 2a). Pair-wise comparisons showed that complexity levels 3–6 were rated as significantly more complex than levels 1 and 2 (all *P*<0.001) (see figure 2a). In addition, the pair-wise comparisons indicated that levels 1 and 2 were not different in their overall perceived complexity (all *p* = 1.000), or levels 3–6 (all *p* = 1.000) (see figure 2a). Musical expertise also had a significant negative effect on ratings (*F*_1, 100_ = 12.36, *p* = 0.002): women with more years of formal musical training gave lower complexity ratings. Age (*F*_1, 20_ = 0.76, *p* = 0.389) and cycle day (*F*_1, 20_ = 1.32, *p* = 0.265) had no effect on women's complexity ratings.

### Liking ratings

The complexity ratings allowed us to create a dichotomous variable for the analysis of liking ratings versus musical complexity; grouping levels 1 and 2 together in a low complexity condition, and levels 3–6 in a high complexity condition. Our results showed that women preferred more complex music (*F*_1, 60_ = 12.86, *p* = 0.001) (see figure 2b); however, no interaction effect between reproductive stage and complexity ratings was observed (*F*_1, 60_ = 0.39, *p* = 0.537), indicating that women displayed the same response pattern across reproductive stages. These findings do not support our hypothesis that women have a heightened preference for more complex music around ovulation (see figure 2b). Reproductive stage had no separate affect on liking ratings (*F*_1, 60_ = 1.69, *p* = 198), nor did age (*F*_1, 20_ = 1.16, *p* = 293) or musical expertise (*F*_1, 20_ = 0.19, *p* = 671).

## Discussion

In this study we found that women have an overall preference for more complex music, replicating the results of previous studies on men and women in which preferences for increased music complexity were observed [18]–[23], [25], [27]. However, we found no evidence that women's preference for more complex music increased when conception was most likely. Consequently, our findings are not consistent with the hypothesis that a heightened female preference for more complex music around ovulation played a role in the evolution of music.

### Complexity ratings

Our results revealed that perceived complexity was mainly affected by the change from isochronous (levels 1 and 2) to non-isochronous sequences (levels 3–6), indicating that complexity is primarily generated by rhythmic variability in our experiment. Interestingly, women with more years of formal musical training gave lower complexity ratings, suggesting that musical expertise moderates how complex the stimuli sound to female listeners. We were somewhat surprised, however, that increasing the potential note range and introducing melodic syncopation (notes produced off the beat) did not significantly increase perceived complexity, as it has done in other studies [20], [22]. It is noteworthy though, that increasing pitch range did consistently raise complexity ratings (levels 2, 4 and 6 compared to 1, 3 and 5: see figure 2a), and also that level 6 received the highest mean rating scores (see figure 2a). Indeed, the shift to non-isochronous sequences may have masked any subtler affect that increasing pitch range and introducing melodic syncopation might have revealed, leading to an apparent asymptote of complexity ratings across levels 3–6.

Taken together then, these results indicate that increasing potential note durations had a much greater effect on women's perceived complexity judgements than increasing pitch range. Crucially though, women did perceive our computer-generated musical stimuli as differing in complexity and hence, we were able to group our stimuli into high and low complexity conditions to examine whether women gave their highest preference ratings for musical pieces perceived as being more complex around ovulation (the primary aim of the current study).

### Liking ratings

Our failure to find a cyclic effect on musical complexity based preferences is difficult to attribute to an inadequate sample size because the *p* level of the interaction effect did not approach significance, making it unlikely that reducing the error variance by increasing sample size would detect an effect that we failed to find. Furthermore, the use of ovulation predictor kits means we could not have failed to test subjects when the likelihood of conception was high, and our stimuli were judged as differing in complexity and thus, appear to be well suited for revealing any cycle-based shifts in complexity preferences. In addition, the use of artificial music compositions and a within-subject design also allowed us to limit the effect of a subject's expertise, familiarity or liking for a given musical style as possible confounding factors [19], [26].

Although we did not find a specific cyclic shift in preferences for more complex music, it is important to note that our findings do not rule out the possibility that ancestral women used the ability of performers to produce complex music as a criteria for mate choice [8], [9]. In mating systems where males contribute little but their genes to offspring, females are expected to choose males using traits that reliably indicate their genetic quality, in order to obtain the indirect benefits of “good genes” for their offspring [8], [34]. Since females only benefit from associating with these individuals when they can conceive, preferences for good genes indicators are expected to emerge during the time of peak conception [15], [35]. However, if the ability to produce complex musical sounds reflects skills valued in long-term mates, such as the ability to provide food and shelter, we might not expect to find the emergence of a preference/or a heightened preference during peak fertility as predicted by ‘good genes’ theories of sexual selection. Furthermore, music's current functions might well differ from those that were operative when it evolved [2], [3]. For instance, music may have originated as a by-product of spoken language [4] and then been co-opted as a sexual signal, or vice versa [8].

It is also noteworthy that we did not consider all aspects of musical complexity in the current study. For example, enhanced chordal and timbral complexity might be considered an indicator of a composer's increased creative ability, a trait shown to be preferred by women over wealth in short-term sexual partners [36]. Other potential indices of a composer's quality may be reflected in intonation and emotional expressivity [37], and future studies could also explore these possibilities. In addition, our study did not directly link compositions differing in complexity with actual performers. Instead, our aim was to reveal a female bias for more complex music around ovulation that could have been exploited by men during sexual courtship.

Accordingly, we suggest that future studies present musical pieces differing in complexity to women and explicitly ask them to choose which *performer*/*composer* they would prefer as a long-term partner versus a short-term sexual partner. Brain-imaging studies could also be used to detect subtle preferential responses to stimuli that may reveal vestiges of sexual selection for specific musical constructs [37]. Furthermore, given the prevalence of vocal music in human culture [38], future work should also examine how women's preferences for vocal stimuli differing in complexity vary across the reproductive cycle. Preferences such as these may also interact with documented preferences for vocal characteristics [39]–[41] that signal heritable characteristics of males, such as their body size [42] and testosterone levels [43], which are potentially important in mate choice contexts. A role for music in sexual courtship has considerable intuitive appeal [8], [9], [12] but, as yet, no empirical backing. Research along these lines will allow female preferences for indicators of potential direct versus indirect genetic benefits to be distinguished, providing a clearer picture of any sexual selection pressures acting on this universal aspect of human culture.

## Supporting Information

Audio S1 **An example of a musical sequence representing complexity level 1.**
(AIF)

Audio S2 **An example of a musical sequence representing complexity level 2.**
(AIF)

Audio S3 **An example of a musical sequence representing complexity level 3.**
(AIF)

Audio S4 **An example of a musical sequence representing complexity level 4.**
(AIF)

Audio S5 **An example of a musical sequence representing complexity level 5.**
(AIF)

Audio S6 **An example of a musical sequence representing complexity level 6.**
(AIF)
